# PROGNOSTIC FACTORS OF LIVER TRANSPLANTATION FOR ACUTE-ON-CHRONIC LIVER FAILURE

**DOI:** 10.1590/0102-672020230061e1779

**Published:** 2023-12-08

**Authors:** Jane CRONST, Lucas PREDIGER, Marcelo Abreu PINTO, Julia FERRAZ, Angelo Zamban de MATTOS, Mario Reis ALVARES-DA-SILVA, Cleber Rosito Pinto KRUEL, Marcio Fernandes CHEDID

**Affiliations:** 1 Universidade Federal do Rio Grande do Sul, Porto Alegre University Hospital, Graduate Program in Surgical Sciences – Porto Alegre (RS), Brazil;; 2 Universidade Federal do Rio Grande do Sul, Porto Alegre University Hospital, Hepatobiliary Surgery and Liver Transplantation Unit – Porto Alegre (RS), Brazil;; 3 Feevale School of Medicine, Medical Sciences – Novo Hamburgo (RS), Brazil;; 4 Santa Casa Hospital Complex – Porto Alegre (RS), Brazil.

**Keywords:** Liver Transplantation, Liver Cirrhosis, Liver Failure, Acute, Gastroenterology, Survival, Transplante de Fígado, Cirrose Hepática, Falência Hepática Aguda, Gastroenterologia, Sobrevida

## Abstract

**BACKGROUND:**

Liver transplantation (LT) is the only treatment that can provide long-term survival for patients with acute-on-chronic liver failure (ACLF). Although several studies identify prognostic factors for patients in ACLF who do not undergo LT, there is scarce literature about prognostic factors after LT in this population.

**AIM:**

Evaluate outcomes of ACLF patients undergoing LT, studying prognostic factors related to 1-year and 90 days post-LT.

**METHODS:**

Patients with ACLF undergoing LT between January 2005 and April 2021 were included. Variables such as chronic liver failure consortium (CLIF-C) ACLF values and ACLF grades were compared with the outcomes.

**RESULTS:**

The ACLF survival of patients (n=25) post-LT at 90 days, 1, 3, 5 and 7 years, was 80, 76, 59.5, 54.1 and 54.1% versus 86.3, 79.4, 72.6, 66.5 and 61.2% for patients undergoing LT for other indications (n=344), (p=0.525). There was no statistical difference for mortality at 01 year and 90 days among patients with the three ACLF grades (ACLF-1 vs. ACLF-2 vs. ACLF-3) undergoing LT, as well as when compared to non-ACLF patients. CLIF-C ACLF score was not related to death outcomes. None of the other studied variables proved to be independent predictors of mortality at 90 days, 1 year, or overall.

**CONCLUSIONS:**

LT conferred long-term survival to most transplant patients. None of the studied variables proved to be a prognostic factor associated with post-LT survival outcomes for patients with ACLF. Additional studies are recommended to clarify the prognostic factors of post-LT survival in patients with ACLF.

## RESUMO

**RACIONAL:**

O transplante hepático (TH) é o único tratamento a proporcionar sobrevida a longo prazo para pacientes com “acute-on-chronic liver failure” (ACLF). Vários estudos identificaram fatores prognósticos para pacientes em ACLF que não realizam TH, porém há poucos dados na literatura sobre fatores prognósticos nessa população transplantada.

**OBJETIVOS:**

Avaliar desfechos de pacientes ACLF submetidos a TH, e seus preditores de mortalidade.

**MÉTODOS:**

Foram avaliados pacientes em ACLF submetidos a TH entre janeiro de 2005 e abril de 2021. Variáveis como valores CLIF-C ACLF e pontuação no ACLF foram comparadas com os desfechos.

**RESULTADOS:**

A sobrevida de ACLF pós TH de pacientes (n=25) em 90 dias, 1, 3, 5 e 7 anos, foi de 80, 76, 59,5, 54,1 e 54,1% versus 86,3, 79,4, 72,6, 66,5 e 61,2% para pacientes submetidos a TH por outras indicações (n=344), (p=0,525). Não houve diferença estatística para mortalidade em 01 ano e 90 dias entre pacientes com os três graus de ACLF (ACLF-1 vs. ACLF-2 vs. ACLF-3), bem como quando comparados a pacientes não ACLF. O escore “chronic liver failure consortium” (CLIF-C) ACLF não se correlacionou com desfechos de óbito. Nenhuma das outras variáveis estudadas mostrou-se preditora independente de mortalidade em 90 dias, após um ano ou global.

**CONCLUSÕES:**

TH conferiu sobrevida em longo prazo à maioria dos pacientes transplantados, semelhante aos pacientes submetidos à TH por outras indicações. Nenhuma das variáveis estudadas mostrou-se fator prognóstico associado a desfechos de sobrevida pós-TH para pacientes com ACLF. Estudos adicionais são necessários para estabelecer fatores prognósticos pós-TH em pacientes com ACLF.

## INTRODUCTION

Acute-on-Chronic Liver Failure (ACLF) is a syndrome defined by acute decompensation of chronic liver disease associated with organ failures. This syndrome is associated with elevated short-term mortality^2-4,7^. Several medical societies from different continents sought to establish a definition of the syndrome, based on aspects like organ failure and disease precipitating factors^10^. Among these definitions, the one established by the Chronic Liver Failure Consortium (CLIF-C) showed better sensibility and performance on mortality prediction, becoming the definition adopted in the present study^9,15,22^.

Data shows that evolution to ACLF occurs in 24-40% of all patients hospitalized for acute decompensation of cirrhosis^12^. Generally, the syndrome triggered by a precipitating event and bacterial infection is the most frequent, followed by active alcohol intake and acute reactivation of B hepatitis^2^. For as much as 40-50% of the patients, no precipitating event is identified^2,12^. In the western world, most patients that evolve to ACLF have chronic liver disease secondarily to alcohol intake or hepatitis C vírus (HCV)^6^.

The 28-days mortality of ACLF was described as 33% by the prospective CANONIC study, ranging from 15% to 80%, depending on the digree of the disease^5^. Even in patients that recover from ACLF without a LT, the estimated mortality for the next 6 months is around 40 to 60%^11^.

LT is generally the first choice for ACLF, since it can treat the syndrome and also eliminate the liver disease. This study aims to analyse the results of LT as a treatment for patients with ACLF. The survival of ACLF patients was also compared to that of all patients who recives LT for other indications in the same period. Predictors of mortality in patients undergoing LT for ACLF were also identified and analyzed

## METHODS

A retrospective cohort study, which includes all adult patients (18 years old or older), submitted to LT for ACLF at HCPA between January 1, 2005, and April 30, 2021. Liver retransplants and combined transplants were excluded (liver and kidney combined transplant, for example), as well as recipients of living donors transplants. The study was approved by the Ethics Committee of the Porto Alegre University Hospital (RS) (number 42306820.0.0000.5327).

All LTs were performed by the Piggyback technique. The immunosuppression was tacrolimus, mycophenolate, and steroids based. Basiliximab induction was provided to kidney injury recipients^13^. Abdominal ultrasonography with color Doppler was periodically performed in all cases to detect hepatic and vascular complications. Oral feeding was early started after extubation in the intensive care unit. In order to avoid heterologous blood transfusion, Cell saver^16^. Fresh frozen plasma, cryoprecipitate, and platelets were administered as needed under thromboelastographic guidance.

The primary outcome was death, which occurred at any time during post-LT follow-up. The secondary outcome was death during the first 90 days post-LT. The patients were followed until death or to the end of the study.

The following pre-LT variables were evaluated: age, gender, Model for End-stage Liver Disease (MELD, MELD-Na), HCV infection, serum albumin, serum total bilirubin, international normalized ratio (INR), serum sodium, serum creatinine, albumin-bilirubin score (ALBI) and ALBI grade, need for dialysis, need for vasoactive drug, encephalopathy degree, ACLF grade, and organs or systems failure. Pre-LT laboratory values were measured 48 hours previous to LT. Regarding to ALBI score, the values were calculated using the following equation: (log^10^ bilirubin [μmol/L] × 0.66) + (albumin [g/L] × - 0.085). Based on ALBI score, patients were classified on three groups according to previously defined cutoff values, resulting in three grades: ALBI grade 1 (=-2.60), grade 2 (-2.60 to=-1.39) and grade 3 (>-1.39).

ACLF diagnoses followed the criteria of CLIF-C^9^:

1. Single renal failure (serum creatinine=2 mg/dL);
2. Single liver, or coagulation, or circulatory, or respiratory failure with serum creatinine between 1.5 to 1.9 mg/dL and/or mild to moderate encephalopathy;
3. Cerebral dysfunction with serum creatinine between 1.5 to 1.9 mg/dL;
4. Two or more organ failures;

The ACLF classification follows the CANONIC study criteria^1^:

1. No ACLF: no organ failure (OF) or a single nonrenal OF without renal dysfunction and cerebral dysfunction.
2. ACLF grade 1 (ACLF-1): single renal failure and single nonrenal OF that is associated with renal dysfunction and/or cerebral dysfunction.
3. ACLF grade 2 (ACLF-2): two OFs of any combination.
4. ACLF grade 3 (ACLF-3): three or more OFs of any combination.

Categorical variables were compared using the chi-square test. Normality test of continuous variables was estimated through the Shapiro-Wilk method. Continuous variables were analysed with Mann-Whitney (U test) (non parametric variables) or T-Test (parametric variables).

Aiming at identifying predictors for main (death at any time during follow-up) and secondary (death on the first 90 days post-LT) outcomes, univariate analyses using Cox proportional regression were performed. In order to identify independent risk factors associated with both primary and secondary outcomes, variables with p-value<0.1 were included in multivariate models using Cox proportional hazards regression model, being considered statistically significant if p<0.05.

Survival was analysed using Kaplan-Meier method, and survival comparison was performed using the log-rank test. For all analyses, p values <0.05 was considered as statistically significant. The analyses were performed using the SPSS 18.0 program for Windows.

## RESULTS

A total of 369 patients were included in this study. 25 of those (6.8%) underwent LT as the treatment for ACLF. 11 patients (44%) were male and 14 female (56%), with the mean age of 52.9 years old (±10.29 years). 9 patients presented ACLF -1 (36%), 8 patients ACLF-2 (32%) and 8 patients had ACLF-3 (32%). All patients had ascites on admission, except for one (Table 1a and Table 1b)

**Table 1a:** Frequency of organ failure in 25 patients.

Renal failure (%)	19 (76)
Liver failure (%)	10 (40)
Circulatory failure (%)	11 (44)
Blood coagulation failure (%)	11 (44)
Brain failure (%)	8 (32)
Respiratory failure (%)	2 (8)

**Table 1b:** Numerical demographic variables for 25 patients in the study.

Numerical demographic variables for non-parametric distribution			
			Hazard ratio [95%CI]

	Total=25 (100%)	Mean±SD	Median+IQR
Age	-	52.9 (±10.29)	-
MELD	-	-	32 (±13)
MELD Na	-	-	32 (±13)
Albumin	-	-	2.8 [IQR_p 25–75_=2,45–3.5]
Sodium	-	-	139.36 [±4]
INR	-	-	2.5 [IQR_p 25–75_=1.82–3.035]
Total bilirubin	-	-	5.6 [IQR_p 25–75_=2–23.8]
V factor (1ºPO)	-	-	66 [IQR_p 25–75_=35–311.25]
**Numerical demographic variables for parametric distribution**			

	**Total=25 (100%)**	**Yes**	**No**
Male gender		11 (44%)	
Number of failing organs/systems		1 organ=11 patients 2 organs=2 patients ≥3 organs=12 patients	
Encephalopathy score (%)		Grade I=10 (40) Grade II=05 (20) Grade III=05 (20) Grade IV=02 (08) No encephalopathy=03 (12)	
Child-Pugh (%)		A–0 (0) B–03 (12) C–22 (88)	
ACLF grade (%)		Grade 1–09 (36) Grade 2–08 (32) Grade 3–08 (32)	
Mechanical ventilation pre-LT		6 (24%)	
ACLF triggering factor, SBP		11 (44%)	
Ascites		24 (96%)	
HCV etiology		17 (68%)	
ALBI		Grade I=3 patients Grade II=5 patients Grade III=17 patients	
Pre-LT sepsis		10 (40%)	
Dialysis		14 (56%)	
Vasoactive drugs		9 (36%)	

CI: confidence interval; SD: standard deviation; IQR: interquartile ratio; MELD: model stage liver disease; MELD Na: model stage Liver Disease sodium; INR: international normalized ratio; PO: postoperative; ACLF: Acute-on-Chronic Liver Failure; SBP: spontaneous bacterial peritonitis; HCV: hepatitis C vírus; ALBI: albumin-bilirubin score; LT: liver transplantation.

The main cause of cirrhosis was HCV infection (n=16, 64%). The median calculated MELD score was (±13) [IQR_p 25–75_=27–40]. The median calculated MELD-Na score was (±13) [IQR_p 25–75_=29–40]. No patient was classified as Child Pugh A, 22 patients were sorted as Child Pugh C (88%) and 3 as Child Pugh B score (12%).

The main precipitating factor of ACLF syndrome was spontaneous bacterial peritonitis (SBP), occurring in 11 patients (44%). The remaining triggering causes were: gastroenteritis (1 patient, 4%), upper gastrointestinal bleeding concomitant with urinary tract infection and pulmonary infection (1 patient, 4%), urinary tract infection only (2 patients, 8%), central venous cateter sepsis (1 patient, 4%), drug-induced hepatitis due to losartan (1 patient, 4%) and Covid-19 (1 patient, 4%). 5 patients (20%) had no identified ACLF triggering factor.

10 patients (40%) presented sepsis at hospital admission. 6 patients required ventilatory support before LT (24%). 2 patients had respiratory failure (8%). 11 patients had circulatory failure (44%). 9 patients received norepinephrine for hemodynamic instability (36%). 19 patients had renal dysfunction or renal failure (76%). 14 patients (56%) required hemodialysis for acute kidney injury before LT. 11 patients had blood coagulation system failure (44%). 22 patients had hepatic encephalopathy (88%). 8 patients had brain dysfunction (32%). 10 patients had liver failure (40%). 19 patients had renal dysfunction or renal failure (76%).

17 patients (68%) were classified as ALBI score 3; 5 patients (20%) as ALBI score 2, and the remaining 3 patients (12%) were classified as ALBI score 01.

The median of total bilirrubin (TB) values was 5.6 mg/dL [IQR_p 25–75_=2–23.8]. The median of albumin values was 2.8 g/dL [IQR_p 25–75_=2.45–3.5]. The median of creatinine values was 1.9 mg/dL [IQR_p 25–75_=1.28–3.25]. The median of INR values was 2.5 [IQR_p 25–75_=1.82–3.03]. The mean of sodium values was 139.36 mEq/L (±4 mEq/L).

The mean of V factor on first post-LT day was 66 [IQR_p 25–75_=35–311.25]. 5 patients evolved to death during hospitalization (20%). 19 patients (76%) reached 90 days of survival. 16 patients reached one year of survival (64%).

The pre-LT hospitalization mean stay was 22 days (±2.9). The median post-LT hospital stay was 33 days [IQR_p 25–75_=21–45.5].

**Figure 1:**
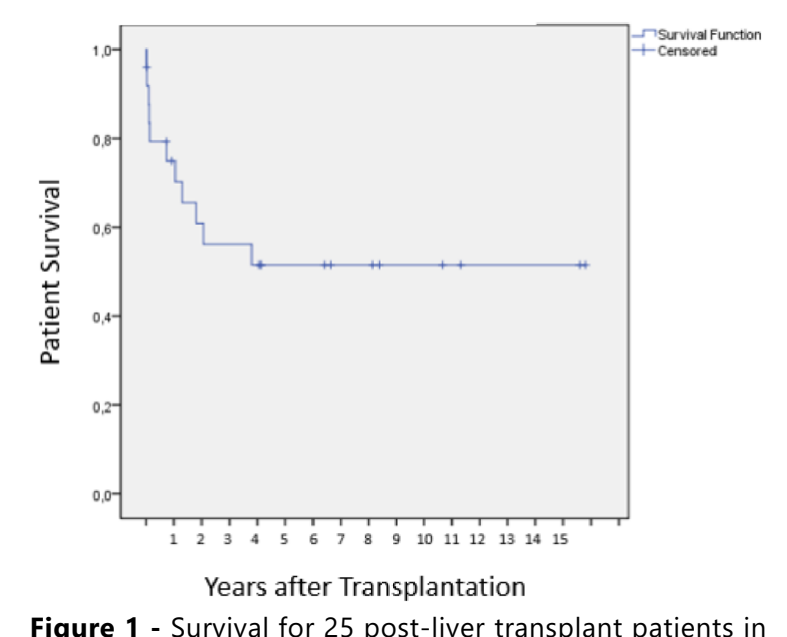
Survival for 25 post-liver transplant patients in Acute-on-Chronic Liver Failure.

**Figure 2:**
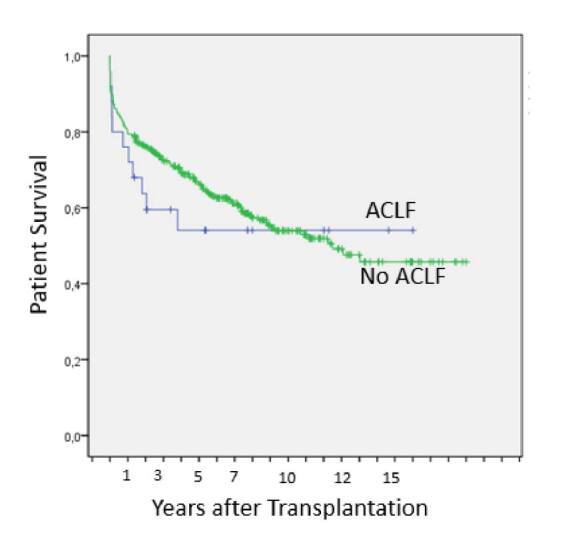
Post-liver transplant survival for 25 consecutive Acute-on-Chronic Liver Failure patients vs. 344 liver transplant patients for other indications in the same period (p=0.525, log-rank).

A survival comparison was performed for 25 patients undergoing LT by ACLF versus 344 patients who underwent LT for other indications. For the ACLF patient group 90-day, 1-, 3-, 5- and 7-year survival was, respectively, 80, 76, 59.5, 54.1 and 54.1% vs. 86.3, 79.4, 72.6, 66.5 and 61.2% for the second group (n=344) (log-rank test, p=0.525) (Breslow test, p=0.288) (Table 2 and Figures 1 and 2).

**Table 2:** Pre and post-LT survival time and hospitalization length for 25 patients.

Pre-LT hospitalization days, mean±SD	22 (±2.9)
Post-LT hospitalization days, median [IQR_p 25-75_]	33 [IQR_p 25–75_=21–45.5]
Post-LT survival 90 days	80%
Post-LT survival 01 year	76%
Post-LT survival 03 years	59.5%
Post-LT survival 05 years	54.1%
Post-LT survival 05 years	54.1%

LT: liver transplantation; SD: standard deviation; IQR: interquartile range.

ACLF: Acute-on-Chronic Liver Failure

An additional analysis evaluated post-LT patients submitted to LT after 2010 (n=274). For ACLF patients, (n=18), 90-day, 1-, 3-, 5- and 7-year survival was, respectively, 88.9, 77.8, 66.2, 57.9 and 57.9% vs. 88.7, 82.4, 74.4, 68.3 and 62.7% for patients undergoing LT for other indications (log-rank test, p=0.643) (Breslow test, p=0.489).

**Figure 3:**
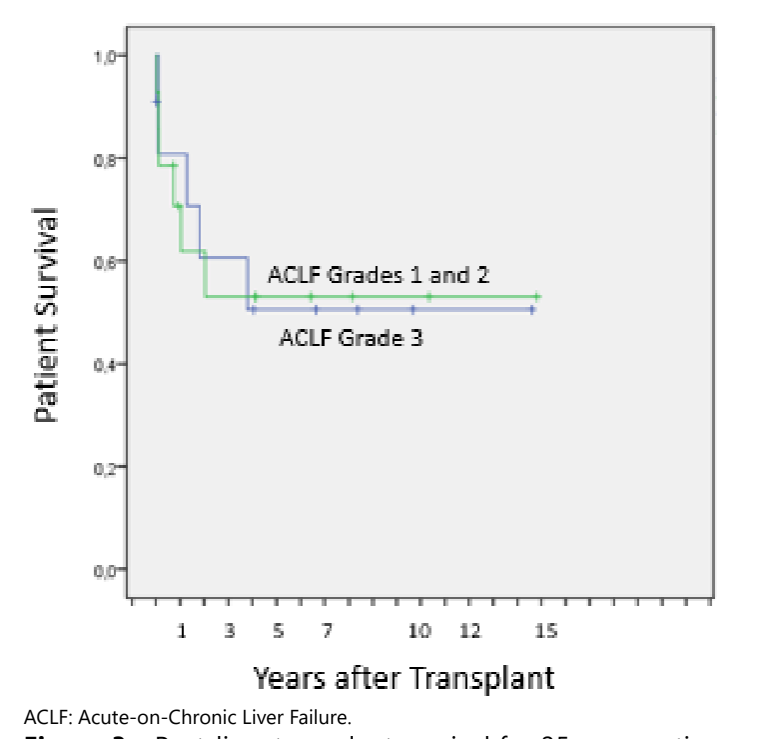
Post-liver transplant survival for 25 consecutive patients undergoing liver transplant for the treatment of Acute-on-Chronic Liver Failure stratified by grade (Grades 1 and 2 as a single group vs. Grade 3) (p=0.981).

An analysis of post-LT survival was performed for the 25 patients who had ACLF stratified by the degree of ACLF (Figure 3). For patients transplanted for ACLF-1 and ACLF-2 combined (n=14), survival at 90 days, 1, 3, 5, and 7 years was 78.6, 70.7, 53, 53 and 53% vs. 80.8, 80.8, 60.6, 50.5 and 50.5% for Grade 3 patients (p=0.981).

ACLF: Acute-on-Chronic Liver Failure.

A univariate analysis by the Cox regression method for overall mortality was performed (Table 3). According to the analysis, none of the studied variables was associated with the outcome. Thus, no multivariate analysis was performed.

**Table 3:** Univariate analysis for mortality-associated factors in 25 patients (Cox regression method).

	Hazard ratio [95%CI]	p-value
Age	0.660 [0.201–2.164]	0.493
Male gender	0.979 [0.264–3.661]	0.979
MELD	1.029 [0.933–1.136]	0.565
MELD Na	1.034 [0.931–1.159]	0.528
HCV Etiology	0.657 [0.191–2.257]	0.504
Total bilirubin	1.004 [0.974–1.029]	0.761
INR	0.987 [0.924–1.056]	0.711
Sodium	1.078 [0.924–1.056]	0.339
Creatinine	0.885 [0.974–1.029]	0.579
ALBI	3.062 [0.658–14.242]	0.154
Serum albumin level	0.849 [0.405–1.778]	0.664
Pre-LT sepsis	0.993 [0.302–3.270]	0.991
Pre-LT dialysis	0.431 [0.130–1.428]	0.164
Pre-LT mechanical ventilation	1.331 [0.352–5.038]	0.673
Pre-LT vasoactive drug use	0.686 [0.181–2.595]	0.579
SBP as a triggering factor	0.526 [0.153–1.802]	0.308
Number of failing organs/systems	1.154 [0.790–1.688]	0.459
CLIF-OF	1.010 [0.831–1.228]	0.917
CLIF-C ACLF score	1.012 [0.949–1.080]	0.711

CI: confidence interval; MELD: model stage liver disease; MELD Na: model stage Liver Disease sodium; HCV: hepatitis C vírus; INR: international normalized ratio; ALBI: Albumin-bilirubin score; LT: liver transplantation; SBP: spontaneous bacterial peritonitis; CLIF-OF: NO Chronic Liver Failure; CLIF-C: chronic liver failure consortium; ACLF: Acute-on-Chronic Liver Failure.

The univariate analysis was performed using the Cox Regression method with a 1-year mortality outcome (Table 4a). According to these analyses, serum albumin [(HR=0.889, IC95% 0.560–1.413 (p=0.836)], total bilirubin [(HR=1.013, IC95% 1.001–1.025 (p=0.027)] and also INR [(HR=1.009, IC95% 1.002–1.016 (p=0.012)] were statistically related to this outcome.

**Table 4a:** Univariate analysis of 1-year post-LT mortality associated factors (Cox regression method).

	Hazard ratio [95%CI]	p-value
Age	1.005 [0.938–1.078]	0.880
Male gender	0.982 [0.264–3.661]	0.979
MELD	0.968 [0.879–1.067]	0.513
MELD Na	0.965 [0.875–1.065]	0.484
HCV etiology	0.513 [0.137–1.923]	0.322
Total bilirubin	1.013 [1.001–1.025]	0.027
INR	1.009 [1.002–1.016]	0.012
Sodium	1.063 [0.899–1.256]	0.474
ALBI	4.683 [0.585–37.499]	0.146
Seruim albumin	1.131 [1.003–1.276]	0.045
Pre-LT sepsis	0.392 [0.081–1.889]	0.243
Pre-LT dialysis	0.381 [0.079–1,272]	0.105
Pre-LT mechanical ventilation	1.095 [0.227–5,284]	0.910
Pre-LT vasoactive drug use	1.025 [0.256–4,102]	0.972
SBP as a triggering factor	0.517 [0.129–2,073]	0.351
Number of failing organs/systems	1.154 [0.790–1,688]	0.459
CLIF OF	1.042 [0.849–1,278]	0.696
CLIF-C ACLF score	1.013 [0.946–1,085]	0.709

CI: confidence interval; MELD: model stage liver disease; MELD Na: model stage Liver Disease sodium; HCV: hepatitis C vírus; INR: international normalized ratio; ALBI: Albumin-bilirubin score; LT: liver transplantation; SBP: spontaneous bacterial peritonitis; CLIF-OF: NO Chronic Liver Failure; CLIF-C: chronic liver failure consortium; ACLF: Acute-on-Chronic Liver Failure;

A multivariate analysis was performed by the Cox regression method with a 1-year mortality outcome (Table 4b). According to the analysis, no variable was associated with the outcome.

**Table 4b:** Multivariate analysis of 1-year mortality-associated factors in 25 patients (Cox regression method).

	Hazard ratio [95%CI]	p-value
International normalized ratio	1.007 [0.997–1.018]	0.180
Serum albumin	0.889 [0.560–1.413]	0.619
Total bilirubin	1.015 [0.969–1.064]	0.525

A comparison between patients that survived 1 year or more and patients that died in the first year of follow-up was performed (time in days – Table 5). For the non-parametic variables, the Mann-Whitney U test was employed; in order to compare the parametric variables, the T-test was performed. The chi-square or Fischer exact test was used for the categorical variables comparison. According to this analysis, none the studied variables was associated with the outcome.

**Table 5:** Comparative analysis between living and dying patients for 1 year.

Non-parametric distribution variables by Mann-Whitney test				
	Total (n=25)	Alive (n=19; 76%)	Dead (n=6; 24%) Hazard ratio [95%CI]	p-value
	Median	Median	Median	
MELD	32 [IQR_p 25–75_=27–40]	32 [IQR_p 25_–_75_=29–40]	30 [IQR_p 25_–_75_=27–37]	0.389
MELD Na	32 [IQR_p 25_–_75_=29–40]	33 [IQR_p 25_–_75_=29.5–40]	30 [IQR_p 25_–_75_=28–37]	0.340
Albumin	2.8 [IQR_p 25_–_75_=2.5–3.3]	2.85 [IQR_p 25_–_75_=2.55–3.65]	2.8 [IQR_p 25_–_75_=2.4–2.8]	0.255
INR	2.5 [IQR_p 25_–_75_=1.82–3.04]	2.48 [IQR_p 25_–_75_=1.68–2.95]	2.75 [IQR_p 25_–_75_=2.31–4.34]	0.308
Total bilirubin	5.6 [IQR_p 25_–_75_=2–23.8]	4.5 [IQR_p 25_–_75_=1.95–24.3]	14 [IQR_p 25_–_75_=1.75–27.15]	0.630
FO number	2 [IQR_p 25_–_75_=1–3]	2.5 [IQR_p 25_–_75_=1–3]	1 [IQR_p 25_–_75_=1–4]	0.571
CLIF OF	12 [IQR_p 25_–_75_=9–14]	12 [IQR_p 25_–_75_=9–14]	10 [IQR_p 25_–_75_=9–13]	0.884
Variables with parametric distribution (mean-CI), t test comparison*				
Male gender	n=11 (44%)	-	-	>99%
Age	52.92 (±10.29)	52.75 (±6.8)	53.2 (±15.04)	0.931
V factor	41.47 (±23.24)	41.59 (±24.75)	41.2 (±21.43)	0.974
CLIF-C ACLF	51 (±10.6)	51 (±9.09)	51.56 (±13.9)	0.930
Sodium	139.36 (±4)	138.94 (±4.25)	140.11(±3.62)	>0.493
**Categorical variables, comparison by χ^2^ test**				
HCV etiology	Total=25 (100%)	n=17 (68%)		0.394
ALBI Grade III	Total=25 (100%)	n=17 (68%)		0.182

* Fisher’s Exact Test comparison; CI: confidence interval; MELD: model stage liver disease; IQR: interquartile range; MELD Na: model stage Liver Disease sodium; INR: international normalized ratio; CLIF-OF: NO Chronic Liver Failure; CLIF-C: chronic liver failure consortium; ACLF: Acute-on-Chronic Liver Failure; HCV: hepatitis C vírus; ALBI: albumin-bilirubin score.

A univariate analysis was performed using the Cox regression method with a 90-day mortality outcome (Table 6a). According to this analysis, albumin [HR=1.139, IC95% 1.010–1.284 (p*=*0.034)] and total bilirubin [HR=1.013, IC95% 1.002–1.025 (p*=*0.027)] were associated with the result.

**Table 6a:** Univariate analysis for mortality-associated factors in 90 days post-LT and 90-day survival time (Cox regression method).

	Hazard ratio [95%CI]	p-value
Age	1.045 [0.957–1.140]	0.327
Male gender	0.72 [0.146–3.578]	0.690
MELD	1.011 [0.894–1.144]	0.859
MELD Na	1.012 [0.889–1.152]	0.854
HCV etiology	0.489 [0.099–2.423]	0.381
Total bilirubin	1.013[1.002–1.025]	0.027
INR	1.007 [0.999–1.015]	0.105
Sodium	1.108 [0.895–1.372]	0.346
ALBI	2.547 [0.297–21.817]	0.394
Serum albumin level	1.139 [1.010–1.284]	0.034
Pre-LT sepsis	0.682 [0.124–3.734]	0.659
Pre-LT dialysis	0.349 [0.064–1.906]	0.224
Pre-LT mechanical ventilation	1.940 [0.354–10.616]	0.445
Pre-LT vasoactive drug use	1.986 [0.400–9.849]	0.401
SBP as a triggering factor	0.243 [0.028–2.084]	0.122
Number of failing organs/systems	1.302 [0.822–2.062]	0.261
CLIF OF	1.167 [0.891–1.529]	0.263
CLIF-C ACLF score	1.053 [0.974–1.138]	0.197

CI: confidence interval; MELD: model for end-stage liver disease; MELD Na: model for end-stage liver disease sodium; HCV: hepatitis C vírus; INR: international normalized ratio; ALBI: Albumin-bilirubin score; LT: liver transplantantion; SBP: spontaneous bacterial peritonitis; CLIF-OF: NO Chronic Liver Failure;; CLIF-C: chronic liver failure consortium; ACLF: Acute-on-Chronic Liver Failure.

A multivariate analysis by the Cox regression method was carried out with the 90-day mortality outcome (Table 6b). According to this analysis, none of the two studied variables were associated with the outcome.

**Table 6b:** Multivariate analysis of associated factors with 90-day post-LT mortality (Cox regression method).

	Hazard ratio [95%CI]	p-value
Serum albumin	0.942 [0.535–1.658]	0.836
Total bilirubin (TB)	1.019 [0.964–1.077]	0.504

CI: confidence interval.

A comparison between patients who survived 90 or more days versus patients who died within the first 90 days was performed (Table 7). For non-parametric variables, the Mann-Whitney U test was used; the T-test was performed for the comparison of parametric variables. For the comparison of categorical variables, the chi-square test or Fisher’s exact test was used. According to this analysis, none of the variables studied was associated with the outcome.

**Table 7:** Comparative analysis between ACLF patients who died in the first 90 days post-LT (n=6) vs. patients who survived 90 days post-LT (n=19).

	All patients (n=25, 100%)	Alive (n=19; 76%)	Dead (n=6; 24%) HR [95%CI]	p-value
		Median	Median	
MELD	32 [IQR_p 25–75_=27–40]	32 [IQR_p 25_–_75_=27.6–38.5]	31 [IQR_p 25_–_75_=27–40]	0.923
MELD Na	32 [IQR_p 25_–_75_=29–40]	32 (29–38.5)	31 [IQR_p 25_–_75_=29–40]	0.923
Albumin	2.8 [IQR_p 25_–_75_=2.45–3.5]	2.8 [IQR_p 25_–_75_=2.55–3.6]	2.7 [IQR_p 25_–_75_=2.4–2.8]	0.406
INR	2.5 [IQR_p 25_–_75_=1.82–3.035]	2.46 [IQR_p 25_–_75_=1.82–2.95]	2.8 [IQR_p 25_–_75_=2.31–4.34]	0.340
Sodium	139.36 [IQR_p 25_–_75_=136–143]	139 [IQR_p 25_–_75_=136–142]	141 [IQR_p 25_–_75_=138–143]	0.264
Total bilirubin	5.6 [IQR_p 25_–_75_=2–23.8]	5.6 [IQR_p 25_–_75_=2.1–22.8]	8.9 [IQR_p 25_–_75_=1.4–36.6]	0.703
OF number	2 [IQR_p 25_–_75_=1–3]	2 [IQR_p 25_–_75_=1–3]	2.5 [IQR_p 25_–_75_=1–5.5]	0.440
CLIF OF	12 5 [IQR_p 25_–_75_=9–14]	10 [IQR_p 25_–_75_=9–14]	12.5 [IQR_p 25_–_75_=9–16.5]	0.396
**Variables with parametric distribution, comparison by t-test**				
	**All patients (n=25, 100%)**	**Mean**	**Mean**	
Male gender	-	11 (44%)	8	0.734
Age	52.9 (±10.29)	51.68 (±9.75)	51.83 (±11.91)	0.295
V factor	185.7(±51.14)	25.9 (±13.8)	44.21 (±23.76)	0.281
CLIF-C ACLF	51.2 (±10.6)	49.79 (±9.25)	55.67 (±13.16)	0.245
**Categorical variables, comparison by the χ^2^ test**				
	**Total=25 (100%)**	**Yes (%)**	**No (%)**	
HCV Etilogy	Total=25 (100)	n=17 (68)	n=8 (32)	0.344
ALBI Grade III	Total=25 (100)	n=17 (68)	n=8 (32)	0.624
Pre-LT sepsis	Total=25 (100)	n=10 (40)	n=15 (60)	0.702
Dialysis	Total=25 (100)	n=14 (56)	n=16 (64)	0.199
Pre-LT MV	Total=25 (100)	n=6 (24)	n= 19 (76)	0.606
Vasoactive drug	Total=25 (100)	n=9 (36)	n=16 (64)	0.630
SBP	Total=25 (100)	n=11 (44)	n=14 (56)	0.122

CI: confidence interval; HR: Hazard ratio; MELD: Model for End-stage Liver Disease; IQR: interquartile range; MELD Na: model for end-stage liver disease sodium; INR: international normalized ratio; CLIF-OF: NO Chronic Liver Failure;; CLIF-C: chronic liver failure consortium; ACLF: Acute-on-Chronic Liver Failure; HCV=hepatitis C vírus; ALBI= albumin-bilirubin score; LT: liver transplantation; MV: mechanical ventilation; SBP: spontaneous bacterial peritonitis.

## DISCUSSION

The 28-day mortality of ACLF patients may reach 80% in 28 days for non-transplanted patients^20^. It is well established that LT is the only treatment capable of providing long-term survival for most patients with ACLF^1^. Several studies have shown that short and long-term survival in LT patients who undergo LT is better than that of non-transplanted patients^2,5,21,22^. Also, based on data from UNOS, a study result found that the probability of surviving while on LT waiting list for more than 30 days for patients with ACLF-3 was less than 10% vs. 90% for patients without ACLF. Therefore, among LT listed patients, those with ACLF died nine times more than those without ACLF.

The present study analyzed 25 cirrhotic patients undergoing LT in ACLF at a single center. Survival rates of 80% at 90 days, 76% at 01 year, 59.5% at 03 years and 64.1% at 05 and 07 years were observed, results comparable to those of the medical literature series^21^. In the present study, the survival difference in transplanted patients by ACLF was about 4.7% at 01 year, 16.6% at 03 years, 15.1% at 05 years, and 9.6% at 07 years. Thus, the lack of stathistically significant difference in overall post-LT survival between ACLF and no-ACLF patients could have occurred because of the relatively small sample size of the cohort analized in this study (n=369).

In this cohort study, after the univariate and multivariate analyses of several potential mortality predictors, no variables related to overall survival or mortality at 90 days and 01 year after LT were identified. In the few studies that evaluated prognostic factors of LT for ACLF, the most important survival predictor was the ACLF grade (a higher number of organ failures was associated with worse outcomes)^17,19^, given that this last group involves patients with three or more organ dysfunctions (multiple organ failures). In the present study, no statistical difference at post-LT survival among the three grades of ACLF was found^17^. Analogous to the present study, some recent studies have not found any difference in post-LT mortality between patients with different grades of ACLF. However, they have pointed to a longer hospitalization and post-LT complications in patients with ACLF grade-3^2^.

Specific scores that can predict mortality in patients with chronic liver disease were evaluated in this study, and some of those scores was specific to ACLF^18,22^. In 2014, Jalan et al. compared MELD, MELD-Na, Child-Pugh and Chronic Liver Failure Consortium (CLIF-C) ACLF scores accuracy at predicting mortality in non-transplanted patients with the ACLF syndrome^9^. Among these scores, CLIF-C ACLF showed the highest accuracy (74.4% for predicting 28-day mortality vs. 0.645% for the MELD score, 0.648% for MELD-Na score and 0.653% for Child-Pugh score). However, there are no specific scores to predict post-LT mortality in patients with ACLF.

In ACLF patients not undergoing LT, CLIF-C ACLF scores above 64 are associated with mortality outcomes so high that they are generally considered unacceptable candidates for LT by some authors, considering the procedure and the institution of intensive measures as futile in these patients^17,20^. However, CLIF-C ACLF was not a poor prognostic factor in the analyses of this series. Furthermore, in the present study, three patients who scored above 64 (with 65, 70 and 78) by the CLIF ACLF score survived the first year after LT, and one of them is alive after 12 years of transplant. All these three patients showed clinical improvement, with recovery from their organ failures before being transplanted. The exact moment to carry out the transplant, particularly for cases as those of ACLF-3 with extreme severity, is extraordinarily difficult. It is believed that clinical improvement would be required for these patients before they could be considered for LT listing. This period of clinical improvement for some patients with ACLF-3 is called “golden window”. The association of the pre-LT golden window with better post-LT results was recently demonstrated by Sundaram et al.^19,20^ These authors evaluated the prognostic factors of mortality at one year after LT for patients in ACLF^19,20^. These authors identified an association between regression from ACLF grade III to ACLF II or I, with a significant mortality reduction in 01 year.

As for the precipitating factor of ACLF, SBP was related to ACLF precipitation in 11 patients (44%), followed by bacterial infections from other sites, in agreement with Western literature data, which include sepsis and pneumonia after SBP^8,17^. On the other hand, none of the patients of these series presented alcohol intake as a precipitating factor, described in the literature as a decompensation frequent cause^17^. Only 2 patients (8%) had a precipitating factor identified as a non-infectious cause (indicated as drug cause and acute biliary pancreatitis). 5 patients had no identified triggering factor (20%), this number being lower than that described in literature, according to which, up to 40% of the time, the cause may not be identified^2,8^. It has also been described that the number of ACLF precipitating events is more important than the type of decompensation as a prognostic factor^17^. In the present study, 3 patients (12%) had more than one precipitating factor identified, of which 2 did not survive the first year after LT.

For all patients in the study, the most common dysfunction was renal injury, present in 19 patients (76%), followed by failure of blood coagulation system and liver failure (both with 11 patients - 44%). When observed in relation to ACLF grades, renal failure was also the most frequent for patients in ACLF grades 1 and 2. In the medical literature, the organ/systems most common failures affecting ACLF patients are, in order: renal (56% of patients), hepatic (44%), coagulation (28%), brain (24%), circulation (17%) and respiratory (9%)^14,23^. In ACLF-3 patients, all systems appear to be highly prevalent.

One limitation of the present study was the sample size. This increases the chances of a type II error occorring. In other words, it may be that, by increasing the number of ACLF cases, some of the variables that were not significant in the univariate analysis could become significant for post-LT death outcomes in ACLF. Another difficulty in carrying out this work was that, as this was a retrospective study arising from the medical records review, the records did not always include the term ACLF, making it difficult to identify patients with the syndrome for inclusion, probably underestimating the number of cases that occured during the study period.

## CONCLUSIONS

LT promotes long-term survival for most ACLF transplanted patients, similar to what occurs to other patients for other indications. None of the analyzed variables in this study was shown to be a prognostic factor associated with post-LT survival in patients with ACLF. Aditional studies evaluating prognostic factors of larger cohorts are warranted to understand the factors related to the prognosis of ACLF patients undergoing LT for ACLF.
